# Identification of Tetraazacyclic Compounds as Novel Potent Inhibitors Antagonizing RORγt Activity and Suppressing Th17 Cell Differentiation

**DOI:** 10.1371/journal.pone.0137711

**Published:** 2015-09-14

**Authors:** Qingfeng Ding, Mei Zhao, Bolan Yu, Chuan Bai, Zhaofeng Huang

**Affiliations:** 1 Institute of Human Virology, Zhongshan School of Medicine, Sun Yat-sen University, Guangzhou, 510080 China; 2 Key Laboratory of Tropical Diseases Control, Sun Yat-sen University, Ministry of Education in China, Guangzhou, 510080 China; 3 Key Laboratory for Major Obstetric Diseases of Guangdong Province, Third Affiliated Hospital of Guangzhou Medical University, Guangzhou, 510150 China; 4 Department of Biochemistry, Zhongshan School of Medicine, Sun Yat-sen University, Guangzhou, 510080 China; University of KwaZulu-Natal, SOUTH AFRICA

## Abstract

CD4^+^ T-helper cells that produce interleukin-17 (Th17 cells) are characterized as pathological T-helper cells in autoimmune diseases. Differentiation of human and mouse Th17 cells requires a key transcription regulator, retinoic acid receptor-related orphan receptor γt (RORγt), which is a potential therapeutic target for autoimmune diseases. To develop a therapeutic agent for Th17-mediated autoimmune diseases, we have established a high-throughput screening (HTS) assay for candidate screening, in which the luciferase activity in RORγt-LBD positive and negative Jurkat cells were analyzed to evaluate induction of RORγt activity by compounds. This technique was applied to screen a commercially-available drug-like chemical compound library (Enamine) which contains 20155 compounds. The screening identified 17 compounds that can inhibit RORγt function in the HTS screen system. Of these, three tetraazacyclic compounds can potently inhibit RORγt activity, and suppress Th17 differentiation and IL-17 production. These three candidate compounds could significantly attenuate the expression of the *Il17a* by 65%- 90%, and inhibit IL-17A secretion by 47%, 63%, and 74%, respectively. These compounds also exhibited a potent anti-RORγt activity, with EC_50_ values of 0.25 μM, 0.67 μM and 2.6 μM, respectively. Our data demonstrated the feasibility of targeting the RORγt to inhibit Th17 cell differentiation and function with these tetraazacyclic compounds, and the potential to improve the structure of these compounds for autoimmune diseases therapeutics.

## Introduction

Retinoic acid receptor-related orphan receptor γt (RORγt) is an orphan nuclear receptor that displays a canonical domain structure with both highly conserved DNA-binding and ligand-binding domains [1]. The RORγt has been demonstrated to be essential for the expression of Interleukin 17(IL-17 also known as IL-17A) and for the development of Th17 cells [2]. Th17 cells are a subset of CD4+ T cells that have been well known as the major source of IL-17 production [3]. IL-17 is a pro-inflammatory cytokine that is involved in inflammation, tissue damage, and bone loss. Previous research has implicated IL-17 and Th17 cells in several human autoimmune diseases such as rheumatoid arthritis (RA), multiple sclerosis (MS), and inflammatory bowel disease (IBD) [4–6].

In 2006, Ivanov *et al*. showed that retinoic acid-related orphan nuclear receptor γt (RORγt) regulates IL-17 production and dictates Th17 differentiation in mouse. RORγt-deficient T cells are not able to differentiate into Th17 cells [7].In addition, genetic ablation of RORγt protected from EAE autoimmune disease with impaired Th17 differentiation [8]. Others have reported that human Th17 cells also express RORγt that share an 88% sequence homology with the mouse form [9]. Thus, RORγt is thought to be a potential drug target for controlling the Th17-mediated autoimmune diseases [10].

RORγt is composed of multiple functional domains known to be important for transcriptional regulation [11]. The N-terminal domain is a highly conserved DNA-binding domain (DBD) and linked via a hinge region with C-terminal ligand-binding domain (LBD) that contains the ligand-dependent transactivation function. The LBD of RORγt can interact with co-activator or co-repressor complexes after binding of the ligand to regulate target gene transcription [12].

Studies have shown that ligands regulate transactivation function by modulating the conformation of LBD [12]. Agonist ligands initiate the LBD activation function by steadying a defined conformation characterized by a hydrophobic coactivator binding surface [13]. Antagonist ligands interfere with the development of an active LBD conformation and inhibit coactivator recruitment [14, 15]. Previous research has found that exogenous small molecules such as digoxin and ursolic acidto can act as antagonist ligands for RORγt [14, 16]. However, the utility of these specific compounds as candidates for further development is limited, as digoxin displays undesirable side effects, and ursolic acid acts on other nuclear receptors [17, 18].

To develop a highly potent therapeutic agent with low toxicity for autoimmune diseases, we established a high-throughput based reporter assay system for screening chemical compounds that can inhibit RORγt-LBD activity. In this study, we identified three tetraazacyclic compounds as novel potent inhibitors that can antagonize RORγt activity and suppress Th17 cell differentiation and IL-17 production.

## Materials and Methods

### Mice

12-week-old C57BL/6 mice were purchased from the Laboratory Animal Center of Sun Yat-Sen University (Guangzhou, China). All mice were maintained in a specific pathogen-free environment in accordance with institutional protocol. All procedures were reviewed and approved by the Laboratory Animal Welfare and Ethics Committee of the Zhongshan School of Medicine, Sun Yat-Sen University.

### Plasmids

The pGL4.31[luc2P/GAL4UAS/Hygro] reporter plasmid and the pBIND vector were purchased from Promega (USA). The fragment of IRES-GFP was first inserted into NotI sites of pBIND. Human RORγtLBD was amplified from cDNA of human PBMC (cDNA is provided by Dr. Hui Zhang [19]) and then cloned into the pBIND-IRES-GFP to generate the fusion sequence Gal4DBD-RORγtLBD-IRES-GFP. The primers that were used to construct the pBIND-Gal4DBD-RORγtLBD-IRES-GFP plasmid are shown as follows: RORγtLBD forward 5’- AACTAGGATCCGAAACCGATGCCAGCACTGC-3’, reverse 5’- AACTAGGATCCGCCTGCTGACAGAAAGCCA -3’.

### Cell Culture

HEK293T cells were maintained in DMEM high-glucose medium (Invitrogen, USA) supplemented with 10% fetal bovine serum (FBS; Hyclone, USA) and 100 U/mL penicillin/streptomycin. Jurkat cells and mouse splenocytes were cultured in RPMI1640 medium (Invitrogen, USA) supplemented with 10%FBS, 2mM glutamine, 1mM sodium pyruvate, 50μM 2-ME, 100U/ml penicillin/streptomycin. All cells were incubated at 37°C in a humidified atmosphere under 5% CO_2_.

### Establishment of the 293T-RORγt and Jurkat-RORγt reporter cell lines

**Fig 1:**
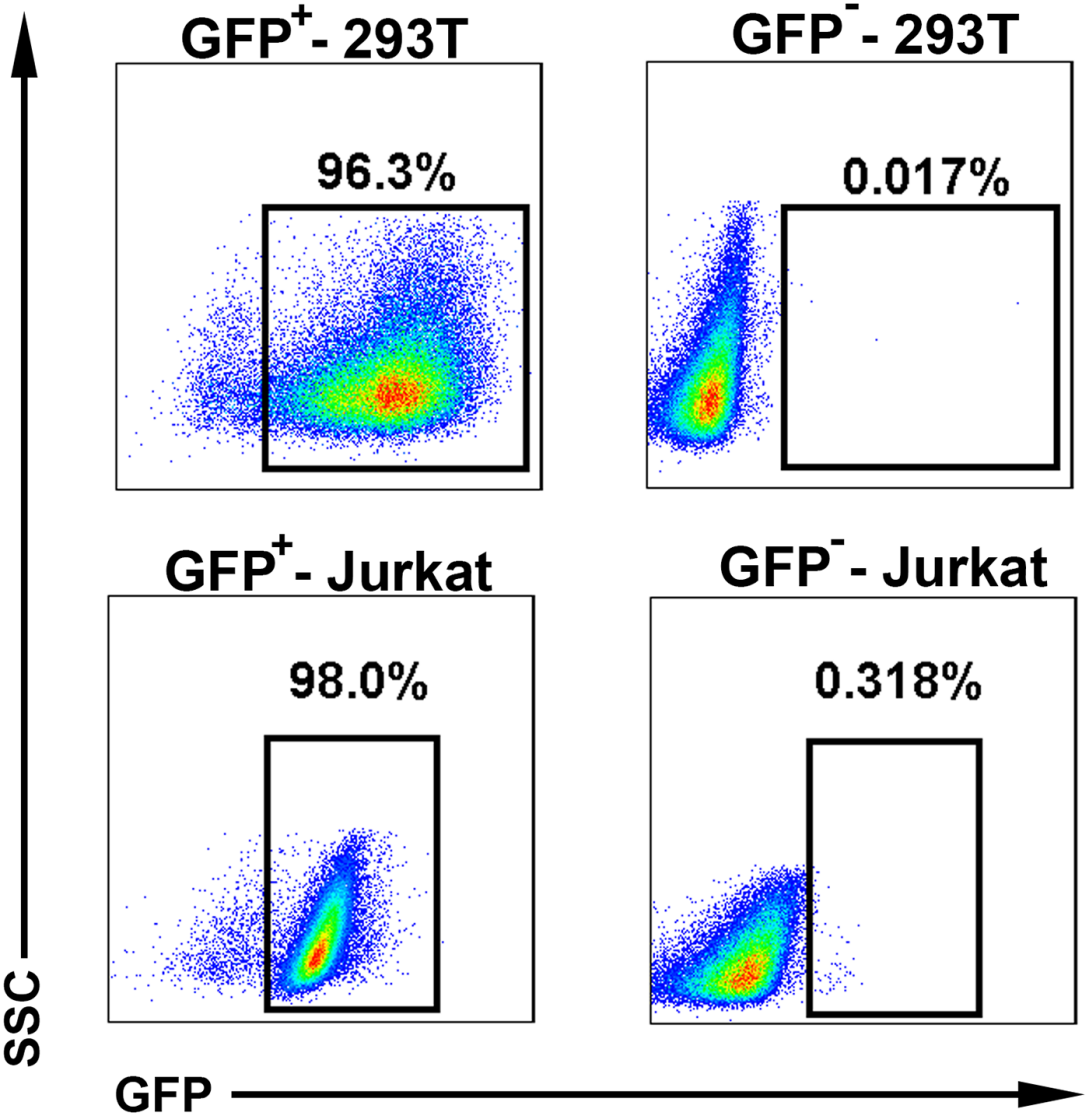
Establishment of the 293T-RORγt and Jurkat-RORγt reporter cell lines. The pGL4.31 reporter plasmids were transfected into 293T cells. The transfected cells were then selected in medium containing hygromycin B to obtain a stable clone which express pGL4.31 reporter gene. Then pBIND-RORγtLBD-IRES-GFP plasmids were also transfected into the stable clone. The second transfected cells were sorted by GFP. GFP^+^-293T stable cell purity was determined >96% by flow cytometry analysis, which yielded a RORγt^+^-293T (RORγt-LBD positive 293T cells) stable reporter cell line, and GFP^-^-293T stable cells were also sorted and used as a RORγt negative control cell line (Fig 1 top panel). Similar to this procedure, Jurkat cells were also transfected with pGL4.31 reporter plasmids by electroporation and selected in RPMI 1640 medium containing hygromycin B. And then the PBIND-RORγtLBD-IRES-GFP plasmids were transfected into the stable Jurkat clone and were sorted by GFP. GFP^+^-Jurkat stable cell purity was determined >96% by flow cytometry analysis and GFP^-^-Jurkat stable cells were also sorted (Fig 1 bottom panel).

The 4 μg pGL4.31 reporter plasmids (promega, USA) were transfected into 293T cells (1 x 10^6^) with Lipofectamin^TM^ 2000 (Invitrogen, USA) according to the manufacturer’s instructions. The transfected cells were then selected in medium containing 100 μg/mL hygromycin B (Roche, USA) for 3 weeks to obtain a stable clone which express pGL4.31 reporter gene. Then 4 μg pBIND-RORγtLBD-IRES-GFP plasmids were also transfected into the stable clone. The second transfected cells were sorted by Green Fluorescent Protein (GFP) [20]. Through this process, we obtained the 293T-RORγt stable reporter cell line. GFP^+^-293T stable cell purity was determined >96% by flow cytometry analysis, which yielded a RORγt^+^-293T (RORγt-LBD positive 293T cells) stable reporter cell line, and GFP^-^-293T stable cells were also sorted and used as a RORγt negative control cell line (Fig 1).

Similar to the above procedure, to obtain the needed Jurkat-RORγt reporter cell lines, a total of 1x10^7^ Jurkat cells were transfected with 20 μg pGL4.31 reporter plasmids by electroporation (Lonza, Swiss) according to the manufacturer’s instructions. The transfected Jurkat cells were also selected in RPMI 1640 medium containing 200 μg/mL hygromycin B. And then the 20 μg PBIND-RORγtLBD-IRES-GFP plasmids were also transfected into the stable Jurkat clone and were sorted by GFP. GFP^+^-Jurkat stable cell purity was determined >96% by flow cytometry analysis and GFP^-^-Jurkat stable cells were also sorted (Fig 1).

### High-Throughput Screening (HTS)

RORγt^+^-Jurkat stable cells (2x10^4^) were seeded into 96-well round-bottom plates (Corning, Costar)and incubated in RPMI1640 complete medium overnight. At initial round screening, drug-like set compounds (Enamine, Monmouth Jct, NJ) were added using a Tecan Freedom EVO150 (Tecan, Männedorf, Schweiz) with a final concentration of 50 μM, which used by other research [21]. 50 μM concentration is pretty high in drug screening and always raises many false positive. To reduce these false positive, we reduced the compounds concentration to 5 μM in the second round validation screening. Column 12 of each plate received only DMSO instead of any compounds as a negative control. In addition, column 1 of each plate was treated with 100 nM PMA (Sigma-Aldrich, USA) as a positive control. 6 hours later, the cells were then lysed and assayed for luciferase activity (Promega, USA) following the manufacturer’s instructions.

### Inhibition of RORγt activity in 293T cells

RORγt^+^-293T stable cells (1x10^4^) were seeded into 96-well plates (Corning, Costar)and incubated in DMEM complete medium overnight. Then the candidates were added with a final concentration of 5 μM. Negative control (DMSO) and positive control were carried on as HTS. 6 hours later, the cells were then lysed and assayed for luciferase activity (Promega, USA) following the manufacturer’s instructions.

### T Cell Differentiation In Vitro

CD4^+^CD25^-^ T cells were isolated from spleens of 12-week-old C57BL/6 as follows: single-cell suspensions were made by crushing the spleen through a cell strainer, and red blood cells (RBCs) were lysed with an RBC lysis buffer. CD4^+^ T cells were then purified using MACS magnetic cell column with a CD4^+^ T cell isolation kit (Miltenyi, Biotec) according to the manufacturer’s protocol. Isolated cells were fluorescently stained with PE-conjugated anti-mouse TCR-βAb, PE-Cy7-conjugated anti-mouse CD4 Ab and APC-conjugated anti-mouse CD25 Ab (BD Bioscience, USA) and analyzed by flow cytometry.

The isolated CD4^+^CD25^-^ T cells (3x10^6^) were cultured under Th17 conditions. Previous studies have shown IL-17A mRNA expression and IL-17A secretion both robustly upregulated after 5 days of polarized differentiation, and the results indicated that it is a good time point for measurement both of mRNA expression and cytokine secretion at day 5 [22]. Specifically, the isolated CD4^+^CD25^-^ T cells (3x10^6^) were stimulated (in 6-well plates) with 5 μg/ml plate-bound anti-CD3e antibody (eBioscience, USA) and 2 μg/ml soluble anti-CD28 Ab (eBioscience, USA) for 24 hours[23]. Then the activated cells were collected, resuspended and seeded into 96-well round-bottom plates (2x10^4^) for Th17 differentiation in the presence of 5 ng/ml recombinant human TGF-β, 30 ng/ml recombinant mouse IL-6 (R&D systems) and DMSO as control or hit compounds with a final concentration of 5 μM. The cells were cultured for 2 days. Later, fresh medium was added (100ul/well) containing recombinant human TGF-β, recombinant mouse IL-6 (R&D systems) and the hit compounds as previously introduced for culturing another 2 days.

### cDNA Synthesis and Quantitative PCR

After 5 days of culture, splenocyte total RNA was extracted with TRIzol (Invitrogen, USA). Then about 1000ng total RNA was added to a 20 μL reaction volume for reverse transcriptions by the GoScript Reverse Transcription System (Promega, USA) according to the manufacturer’s instructions, followed by real-time quantification using GoTaq qPCR Master Mix (Promega, USA). Gene expression of mouse *Rorγt*, *Il17a* and *Il17f* was normalized to the expression of *Gapdh*. The following primer sequences were used: *Rorc* forward 5’-TGTAATGTGG CCTACTCCTGCA-3’, reverse 5’-AAACTTGACAGCATCTCGGGA-3’; *Il17a* forward 5’-CTCCAGAAGGCCCTCAGACTAC-3’, reverse 5’-AGCTTTCCCTCCGCATTGACACAG-3’; *Il17f* forward 5’-GAGGATAACACTGTGAGAGTTGAC-3’, reverse 5’- GAGTTCATGGTGCTGTCTTCC-3’; *Gapdh* forward 5’-TGGTGAAGGTCGGTGTGAAC-3’, reverse 5’-CCATGTAGTTGAGGTCAATGAAGG-3’.

### ELISA

After 5 days of culture, the concentration of IL-17A in the supernatant was determined by an ELISA kit according to the manufacturer’s protocol (CUSABIO Systems, China).

### EC_50_ Assay

Jurkat-RORγt^+^ stable cells were seeded into 96-well round-bottom plates (2x10^4^) overnight and incubated with the compounds by 5-fold gradient, with final concentrations from 5μM to 8nM. 6h later, the cells were then lysed and assayed for luciferase activity (Promega, USA) following the manufacturer’s instructions. The values of half-maximal effective concentration (EC_50_) were determined by plotting the logarithm of compound concentration versus relative luciferase activity [15].

### Cell viability Assay

The cell viabilities of Jurkat cells (wild type) after treatment with chemicals were assessed by MTT technique. In brief, wild type Jurkat cells were seeded (2x10^4^) and incubated with the compounds by 5-fold gradient concentrations, as described above. 48h treatment later, MTT (Dimethylthiazolyl-2-5-diphenyltetrazolium bromide) dye solution (Sigma, USA) was added into the 96-well plate and incubated at 37°C for 4 h, MTT is cleaved by live cells to a colored formazan product. After centrifugation (1500rpm, 5min) the supernatant was discarded. 100μl DMSO was added to dissolve the formazan product and gently shaken for 10min. Absorbance at 570 nm wavelength was recorded using a Thermo Scientific Multiskan FC (Thermo Scientific, USA). Each treatment was repeated in quadruplicate. Cell viabilities were defined relative to control cells (DMSO treated). The half-maximal cytotoxic concentration (CC_50_) for each compound was calculated from these dose-response curves with the aid of Graphpad Prism software (GraphPad Software, CA).

### Statistical Analysis

All data are shown as the means ± SEM. Statistical significance was determined by an unpaired t test using GraphPad Prism software 5.0 (GraphPad Software, CA). P values <0.05 were considered to be statistically significant.

## Results

### Validation of the Jurkat-RORγt-LBD and 293T-RORγt-LBD stable reporter cell lines

Researchers have found that PMA-mediated PKC-θ activation dramatically increased IL-17 and RORγt mRNA expression and promoted Th17 differentiation [22]. To confirm the validity of the RORγt reporter system, we added different concentrations of phorbol-12-myristate-13-acetate (PMA) to evaluate both basal and inducible levels of RORγt activity by measuring changes in luciferase activity in Jurkat-RORγt-LBD and 293T-RORγt-LBD stable cell lines over a time course.

**Fig 2:**
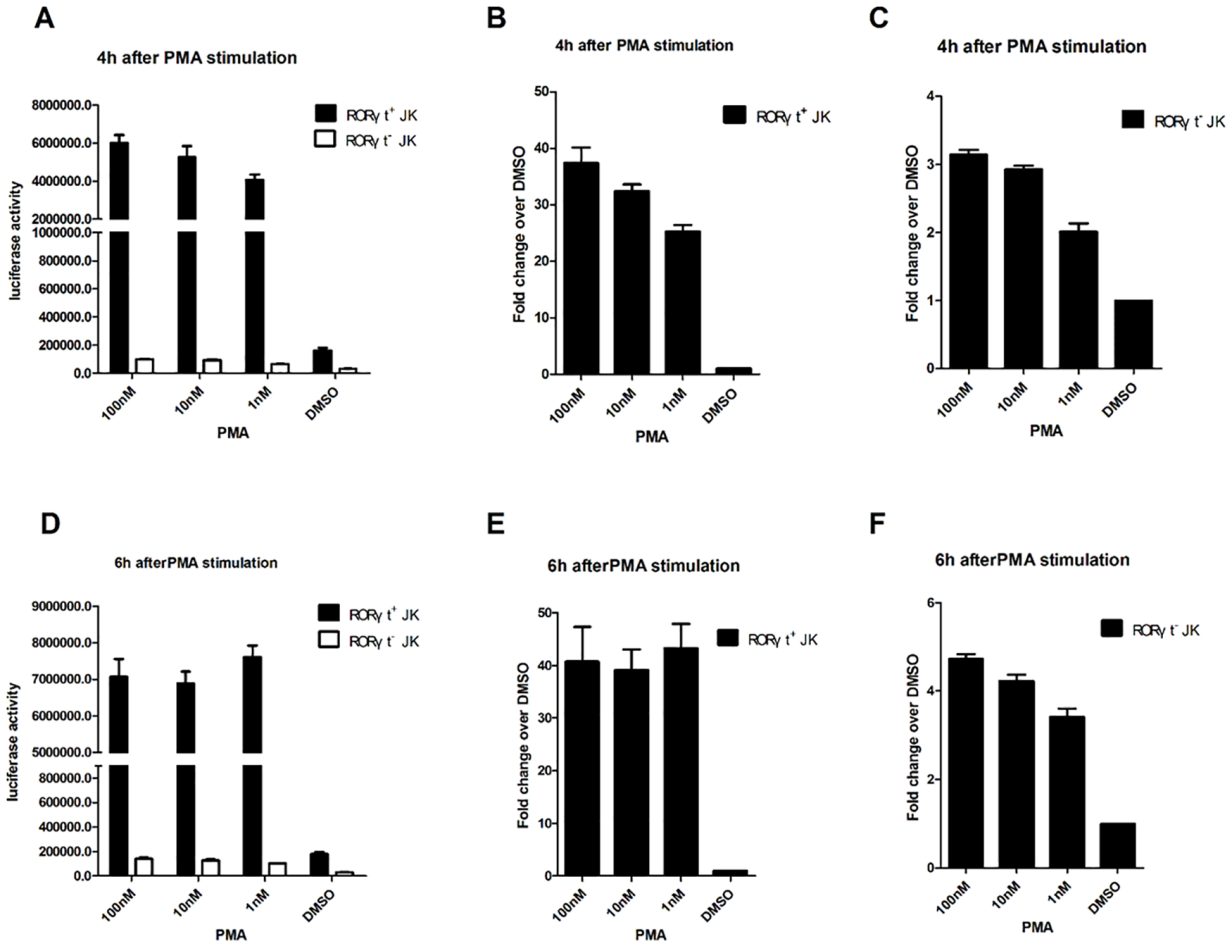
Validation of the Jurkat-RORγt-LBD stable cell lines. RORγt^+^-Jurkat (RORγt-LBD positive Jurkat cells) and RORγt^-^-Jurkat (RORγt-LBD negative Jurkat cells) stable cells (1x10^5^) were seeded onto 12-well plates and incubated in 1 mL RPMI 1640 complete medium overnight. The cells were cultured with different concentrations of PMA (100 nM, 10 nM, 1 nM) or DMSO (vehicle control) for 4hr. The total protein was extracted to determine luciferase activity (A). The fold changes in luciferase activity compared to the DMSO control were determined in RORγt^+^-Jurkat cells and RORγt^-^-Jurkat cells, when the cells were cultured with different concentrations of PMA (100 nM, 10 nM, 1 nM) or DMSO for 4hr (B, C). RORγt^+^ and RORγt^-^-Jurkat cells (1 × 10^5^) were seeded onto 12-well plates and incubated in 1 mL RPMI 1640 complete medium overnight. Then the cells were cultured with different concentrations of PMA (100 nM, 10 nM, 1 nM) or DMSO (vehicle control) for 6 hr, at which point total protein was extracted to determine luciferase activity (D). The fold changes in luciferase activity upon exposure to compounds vs DMSO were determined in RORγt^+^-Jurkat cells and RORγt^-^-Jurkat cells using different concentrations of PMA (100 nM, 10 nM, 1 nM) or DMSO (vehicle control) for 6hr (E, F). The results are shown as means ± SEM.

RORγt^+^-Jurkat (RORγt-LBD positive Jurkat cells) and RORγt^-^-Jurkat (RORγt-LBD negative Jurkat cells) stable cells (1x10^5^) were seeded onto separate 12-well plates and cultured overnight. Cells were then stimulated with different concentrations of PMA (100 nM, 10 nM, 1 nM) or DMSO (negative control) for 4, 6, and 8 hr. Significantly, we found that PMA increased the luciferase reporter activity in RORγt^+^-Jurkat cells in a dose-dependent manner at 4 hr (Fig 2A). Luciferase activity was highest in cells cultured with 100nM PMA, it was at approximately 40-fold over the DMSO control (Fig 2A and 2B). When RORγt^+^-Jurkat cells were cultured for 6 hr, the luciferase activity was nearly identical across the concentrations and increased from control by about 40-fold at various concentrations (Fig 2D and 2E). This increase in fold changes, as assayed by PMA-induced reporter activity, was not observed after 8 hr of treatment (data not shown). Therefore, this 40-fold change may be the highest level of PMA-induced activation in RORγt^+^-Jurkat cells. In contrast, luciferase activity in RORγt^-^-Jurkat cells with different concentrations of PMA exhibited no significant increase (Fig 2C and 2F). Additionally, when cells were cultured in DMSO at all times, the luciferase activity in RORγt^+^-Jurkat was always about 5-fold higher than that in RORγt^-^-Jurkat cells (Fig 2A and 2D). These results were fully in line with expectations in this reporter system, according to the manufacturer’s instructions (Promega, USA). These results validate that the RORγt ^+^-Jurkat cells faithfully responded to the effects of PMA after a 4–6 hr treatment and can be used as a T cell reporter system for RORγt activity.

**Fig 3:**
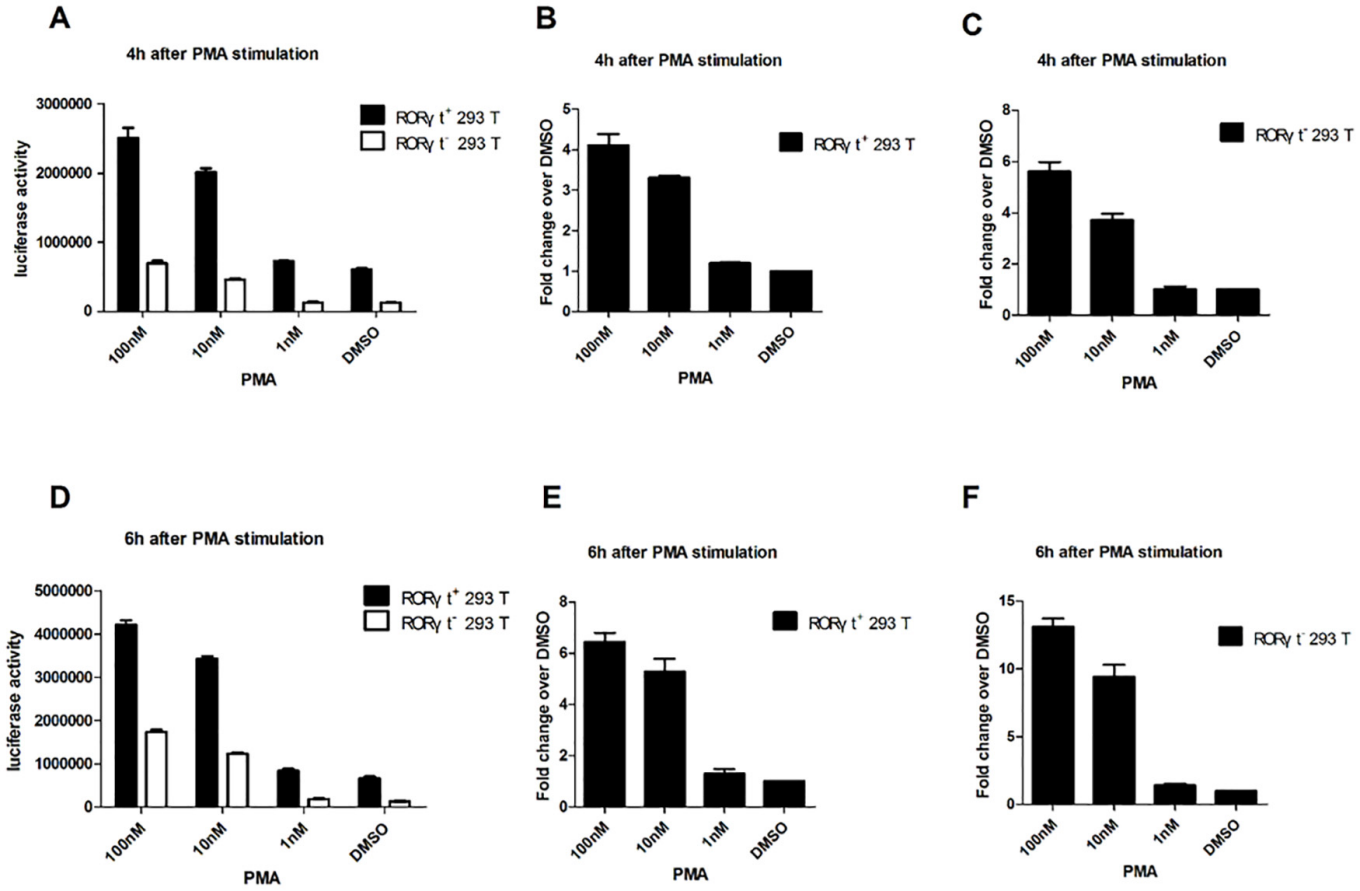
Validation of the 293T-RORγt-LBD stable cell lines. RORγt^+^-293T (RORγt-LBD positive 293T cells) and RORγt^-^-293T (RORγt-LBD negative 293T cells) stable cells (1x10^5^) were seeded onto 12-well plates and incubated in 1 mL DMEM complete medium overnight. Then the cells were cultured with different concentrations of PMA (100 nM, 10 nM, 1 nM) or DMSO for 4h. The total cell proteins were extracted and luciferase activity was determined (A). The fold changes in luciferase activity compared to the DMSO control were determined when the RORγt^+^-293T and RORγt^-^-293T cells were cultured with different concentrations of PMA (100 nM, 10 nM, 1 nM) or DMSO (vehicle control) for 4h (B, C). RORγt^+^-293T and RORγt^-^-293T stable cells (1x10^5^) were seeded onto 12-well plates as above. Then the cells were cultured with different concentrations of PMA (100 nM, 10 nM, 1 nM) or DMSO (vehicle control) for 6h. Total cell proteins were extracted and luciferase activity was determined (D). The fold changes in luciferase activity upon exposure to compounds vs DMSO were determined in RORγt^+^-293T and RORγt^-^-293Tcells using different concentrations of PMA (100 nM, 10 nM, 1 nM) or DMSO (vehicle control) for 6 h (E, F). The results are shown as mean ± SEM.

Similarly, RORγt^+^-293T (RORγt-LBD positive 293T cells) and RORγt^-^-293T (RORγt-LBD negative 293T cells) stable cells were also cultured in the presence of PMA (100 nM, 10 nM, 1 nM) or DMSO (vehicle control) for 4 hr and 6 hr. As observed in the Jurkat cell lines, the basal luciferase activity in RORγt^+^-293T was always about 5 folds higher than in ROR^-^-293T cells (Fig 3A and 3D). Furthermore, PMA increased the luciferase activity of RORγt^+^-293T cells in a dose-dependent manner at 4 hr and 6 hr (Fig 3A and 3D), indicating that the function of the RORγt-LBD in 293T cells was also intact. However, the greatest increase in luciferase activity in RORγt^+^-293T cells (100nM PMA) was only about 4–6 folds greater than that with DMSO exposure at 4 hr and 6 hr (Fig 3B and 3E). Because RORγt is a master transcriptional regulator in T cells, these results confirmed that RORγt has a significantly higher effect on Jurkat cells in response to PMA than that on 293T cells. Thus, this difference can be used as an evaluation marker to denote T cell-specific effects in the screened compounds.

### Identification of candidates by High-throughput Screening (HTS)

We developed a high-throughput screening (HTS) assay using RORγt^+^-Jurkat (RORγt-LBD positive Jurkat cells) stable reporter cells to test the anti-RORγt-LBD activity of small molecules. We used this assay to screen a commercially-available drug-like chemical compound library (Enamine). In the HTS system, the RORγt^+^-Jurkat cells (2x10^4^) were seeded onto 96-well plates and incubated in complete medium overnight, followed by the treatment with compounds at a final concentration of 50 μM for 6 hr during which the luciferase activity was measured. Compounds that showed inhibitory activity on reporter luciferase by more than 50% were incubated again with the cells at a 5 μM concentration for 6 hr in a secondary screen to confirm the previous results. 17 compounds were identified as hit compounds, defined as compounds that can inhibit RORγt function in the HTS screen system.

### Candidate compounds inhibited mouse Th17 cell differentiation

**Fig 4:**
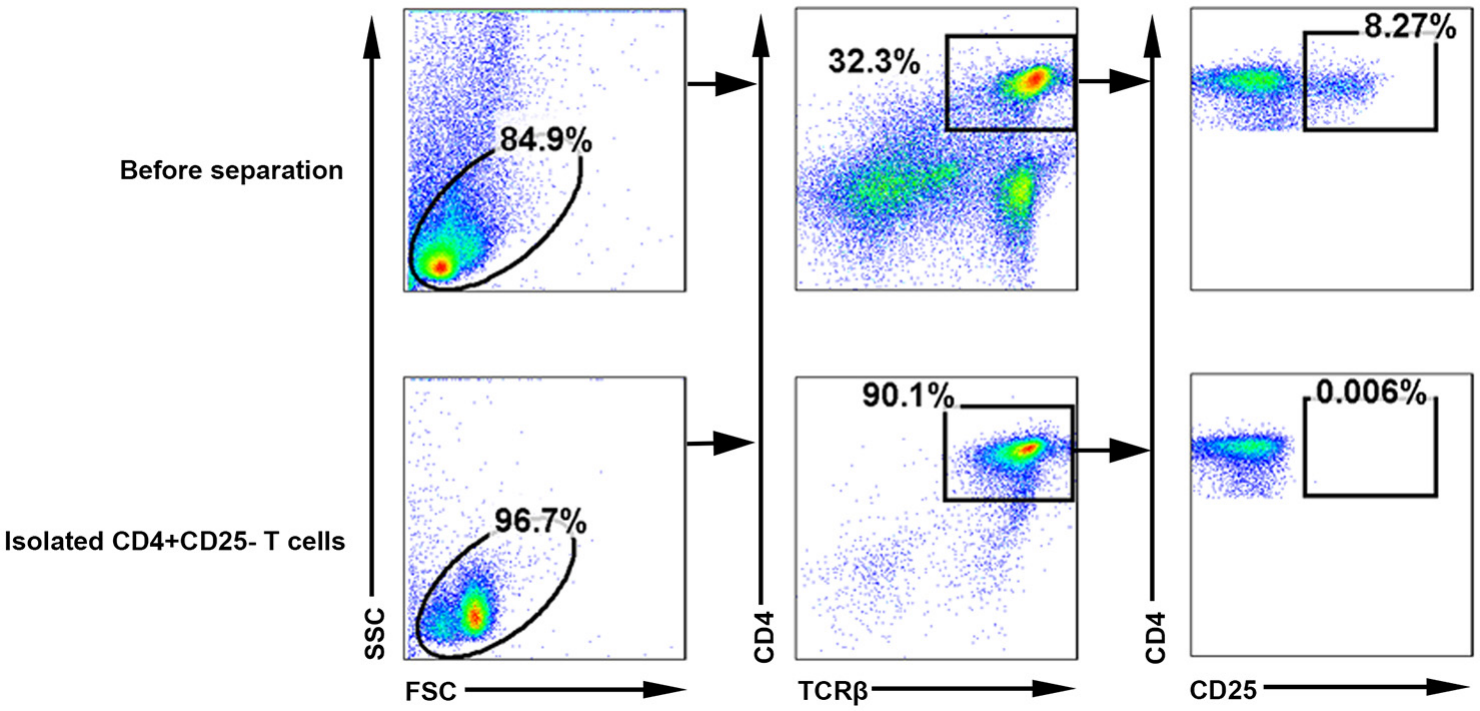
Purification of the CD4^+^CD25^-^ T cells. Single-cell suspensions from spleens of 12-week-old C57BL/6 were made by crushing the spleen through a cell strainer, and red blood cells (RBCs) were lysed with an RBC lysis buffer. CD4^+^ T cells were then purified using MACS magnetic cell column with a CD4^+^ T cell isolation kit. And about 90% cells were CD4^+^ CD25^-^ T cell after isolation. The proportion was significantly higher than before separation, which was only about 32%.

Because RORγt-LBD activity is required for optimal Th17 cell development, we next explored whether the hit compounds identified in the HTS assay could sufficiently inhibit Th17 cell differentiation. CD4^+^CD25^-^ T cells were isolated using MACS magnetic cell column and obtained 90% purity which checked by FACS (Fig 4). Isolated CD4^+^CD25^-^ T cells were cultured under Th17 polarizing conditions (IL-6 and TGF- β) in the presence of the candidates or vehicle control (DMSO) for 4 days.

**Fig 5:**
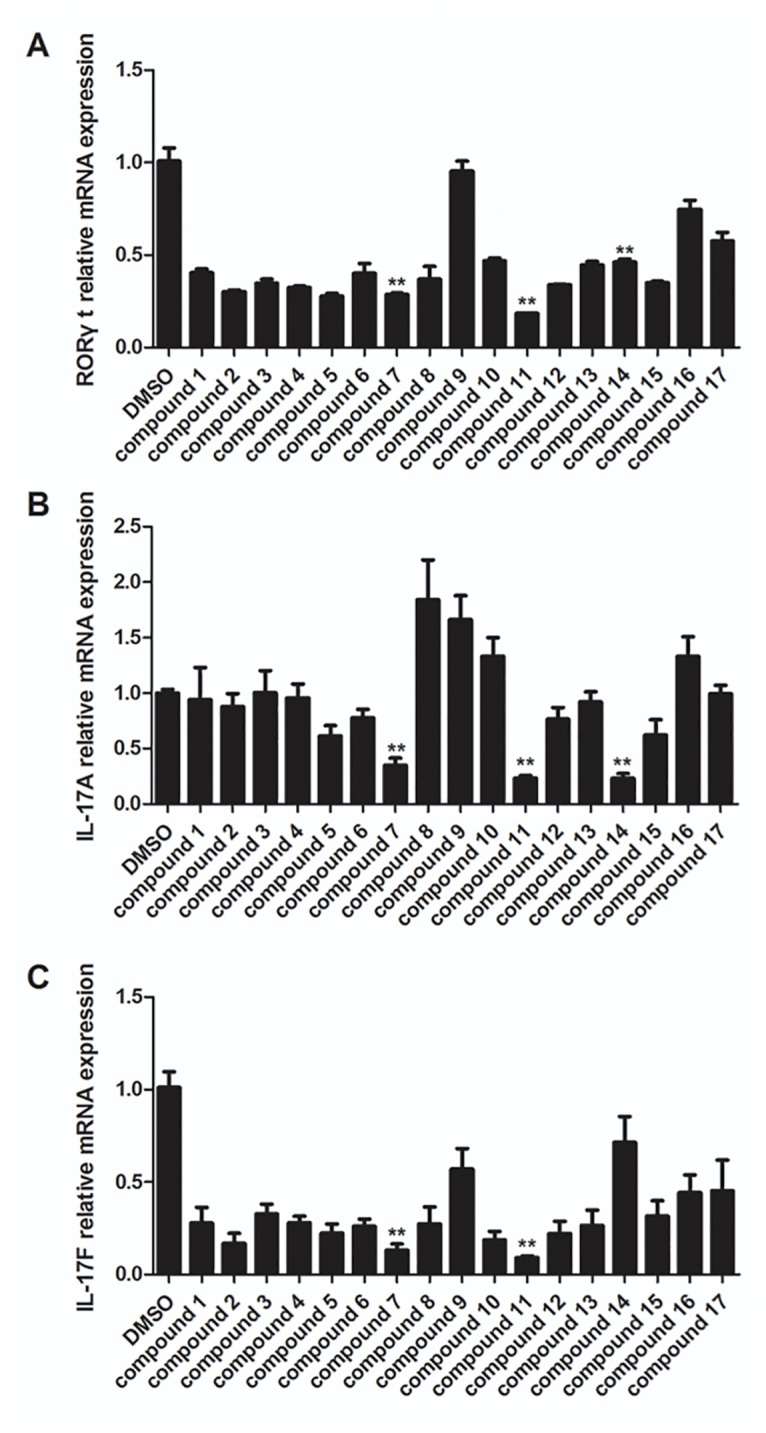
Three candidate compounds inhibit mouse Th17 cell differentiation. CD4^+^CD25^-^ T cells were isolated from spleens of 8–12 week old mice using MACS magnetic cell column with a CD4^+^ T cell isolation kit. CD4^+^CD25^-^ T cells were cultured under Th17 polarizing conditions with vehicle control and hit compounds (5 μM) as described in the methods section. RORγt (A), IL-17A (B) and IL-17F (C) expression was quantified and normalized to GAPDH. The results are shown as mean ± SEM; ** *P*< 0.01.

As expected, the combination of TGF-β and IL-6 increased the mRNA expression of *RORγt*, *Il17a*, and *Il17f* in vehicle-treated cells, and almost all of the candidate compounds inhibited expression of *RORγt* (Fig 5A), whereas only 3 out of the initial 17 compounds (compounds 7, 11 and 14) were able to significantly attenuate the expression of the *Il17a* by 65%-80%, compared with vehicle-treated control group (Fig 5B). However, compound 14 did not significantly inhibited *Il17f* expression, and compounds 7, 11 suppressed the expression of the *Il17f* by 87% and 90%, respectively (Fig 5C). The structures of these three candidate compounds were summarized on Table 1. Interestingly, structural analysis of the resulting compounds indicated that compounds 7, 11 and 14 share a highly similar scaffold. These three candidates are tetraazacyclic compounds which are made of a tetrazolium benzene group and an aromatic heterocyclic group connected by a single sulfur bridge (Table 1).

**Table 1:** The structures of compound 7, 11, and 14.

Compound	Structure name
compound7	N-[[5,6-dimethyl-4-(1-phenyltetrazol-5-yl)sulfanylthieno[3,2-e]pyrimidin-2-yl]methyl]-N-ethylethanamine
compound11	7-chloro-4-[1-(2-methylphenyl)tetrazol-5-yl]sulfanylquinoline
compound14	4-[1-(2,6-dimethylphenyl)tetrazol-5-yl]sulfanylthieno[3,2-d]pyrimidine

### Candidate compounds inhibited IL-17A secretion

**Fig 6:**
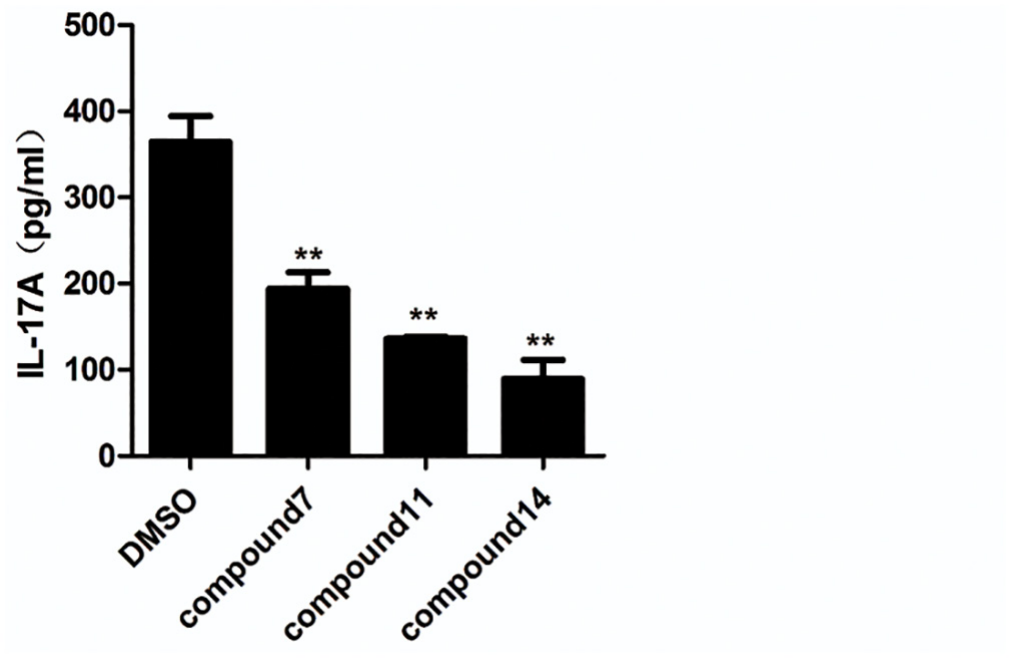
Three tetraazacyclic compounds inhibit IL-17A secretion. CD4^+^ T cells were cultured under Th17 polarizing conditions and the effect on IL-17A secretion in the supernatant was assessed by enzyme-linked immunosorbent assay (ELISA). The concentrations of IL-17A secreted from Th17 cells in presence of tetraazacyclic compounds at 5 μm or DMSO were determined. The results are shown as mean ± SEM; ** *P*< 0.01.

We also assessed whether these three tetraazacyclic compounds could inhibit IL-17A protein secretion. CD4^+^ T cells were cultured under Th17 polarizing conditions and assessed the effect on IL-17A secretion in supernatant by ELISA. The results showed that the three compounds also inhibited IL-17A secretion, consistent with the results of the qPCR (Fig 6). Treatment by compounds 7, 11, and 14 reduced IL-17A concentration to 196 pg/mL, 136 pg/mL, 88 pg/mL (a reduction of 47%, 63%, and 74%), respectively, compared with vehicle-treated control cells (Fig 6).

### EC_50_ and CC_50_ values of the tetraazacyclic compounds

The EC_50_ and CC_50_ of all three compounds were determined to further identify the effects of these three tetraazacyclic compounds. RORγt^+^-Jurkat cells were treated with titrated compounds in a 5-fold gradient, with final concentrations ranging from 5 μM to 8 nM for 6 hr, and a relative luciferase activity was recorded to determine the EC_50_. The CC_50_ values of each compound in Jurkat cells were also determined as previously described in the methods section.

**Fig 7:**
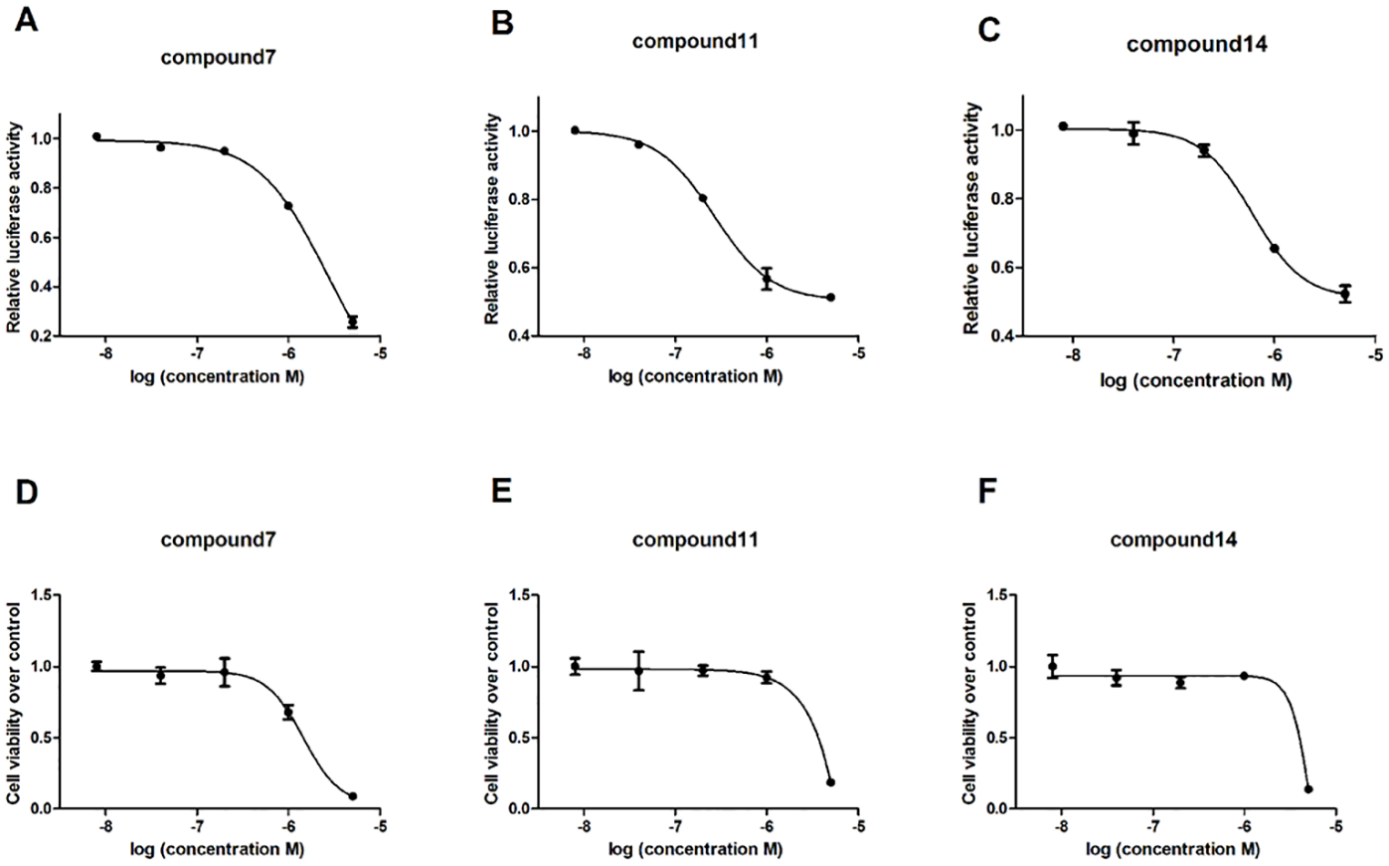
EC_50_ and CC_50_ of novel tetraazacyclic compounds. RORγt^+^-Jurkat cells were seeded onto 96-well round-bottom plates (2x10^4^) overnight and incubated with the compounds titrated at 5-fold gradient final concentrations of 5 μM to 8 nM. 6 h later, relative luciferase activity was recorded to obtain the EC_50_ values of compounds 7, 11 and 14 (A, B, C, respectively). Jurkat cells (wild type) were seeded onto 96-well round-bottom plates (2x10^4^) overnight and incubated with the compounds at 5-fold gradient concentrations. 48 h treatment later, MTT was added onto 96-well plate and incubated at 37°C for 4 h. MTT was cleaved by live cells to a colored formazan product. After centrifugation (1500rpm, 5min), the supernatant was discarded. 100 μl DMSO was added to dissolve the formazan product and the solution was gently shaken for 10 min. Absorbance at 570 nm wavelength was recorded to identify CC_50_ values of compounds 7, 11 and 14 (D, E, F, respectively). The results are shown as mean ± SEM.

Compound 7 exhibited a potent anti-RORγt activity and high cytotoxicity, with EC_50_ and CC_50_ values of 2.6 μM and 1.5 μM, respectively (Fig 7A and 7D). Unlike compound 7, compound 11 and 14 showed higher and more potent suppressive activity with EC_50_ values of 0.25 μM and 0.67 μM, respectively (Fig 7B and 7C). Furthermore, compounds 11 and 14 also showed limited cytotoxicities with CC_50_ values significantly greater than 5 μM (the highest concentration in this assay) (Fig 7E and 7F). The potent inhibitory effect and limited cytotoxicity of compounds 11 and 14 suggest that these two novel inhibitors may be effective anti-RORγt drugs for treatment of auto-immune disease.

### Inhibition of RORγt activity in 293T cells

RORγt ^+^-293T cells were used to test inhibition of RORγt activity by compounds in different cell types. The results can be used as an evaluation marker to denote T cell-specific effects of these tetraazacyclic compounds.

**Fig 8:**
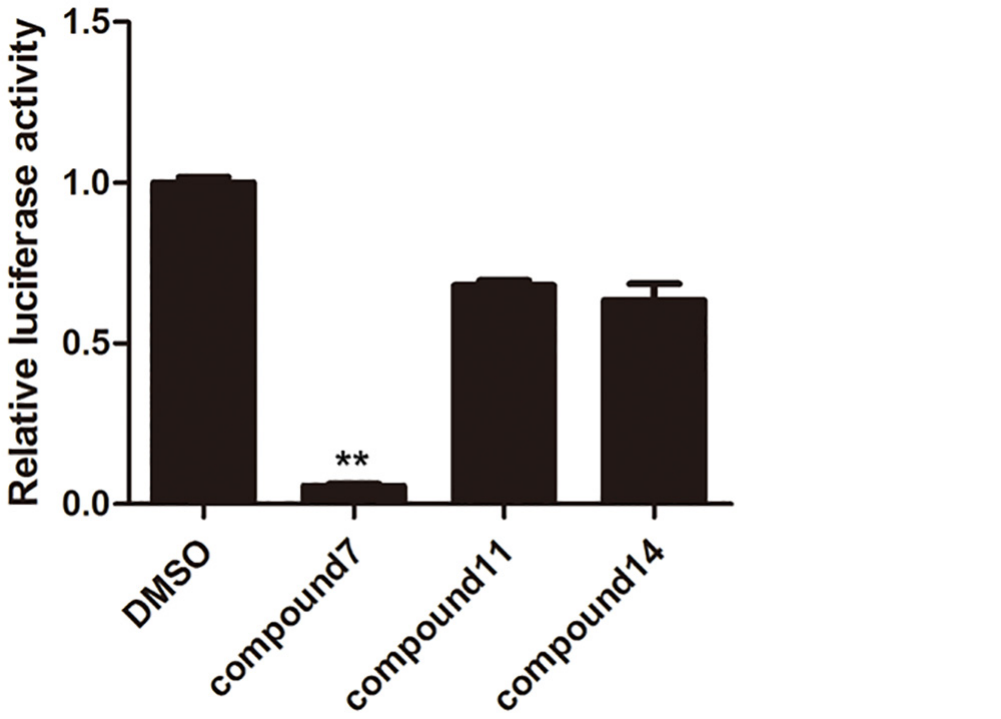
Inhibition of RORγt activity in 293T cells. RORγt^+^-293T stable cells were culture in the presence of hit compounds (5 μM) and DMSO (vehicle control) for 6 h. Then the luciferase activity in RORγt^+^-293T cells were recorded. The results can be used as an evaluation marker to denote T cell-specific effects of these tetraazacyclic compounds. The results are shown as mean ± SEM; ** *P*< 0.001.

Compound 7 at 5 μM inhibited the reporter activity in RORγt^+^-293T cells by 95% (Fig 8), which is significantly more than the 75% observed in RORγt^+^-Jurkat cells compared with vehicle-treated control cells at same concentration (Fig 7A). In contrast, compounds 11 and 14 showed significantly less inhibition in RORγt^+^-293T cells (Fig 8) than in RORγt^+^-Jurkat cells (Fig 7B and 7C). These results indicated that inhibitory activity of the three compounds vary according to the type of T cells.

## Discussion

The essential role of RORγt as a master regulator in Th17 cell differentiation and IL-17 production has been described previously. RORγt is thought to be a promising, novel drug target for the clinical treatment of many autoimmune disorders [15],[24]. Another IL-17 family member, IL-17F, is also abundantly expressed in Th17 cells and has a high sequence homology and functional similarity with IL-17A [15]. Increased level of IL-17F has also been detected in autoimmune cases, suggesting that IL-17F may play a similar role to that of IL-17A [4, 25, 26]. Recently, several reports have shown that digoxin, ursolic acid, and SR1001 can inhibit RORγt activity which in turn can reduce IL-17A secretion and Th17 cell differentiation. Unfortunately, digoxin has severe side effects in clinical therapy, ursolic acid lacks specificity due to interaction with other nuclear receptors [17],[18], SR1001 and its several derivatives exhibited a relatively weak activity in Th17 cell differentiation study [27, 28]. In addition, although few candidates, such as ML209 and Tertiary amide and indole derivatives were discovered as potent RORγt inverse agonists, the clinical applications of them have many challenges in safety hypothesis in humans[29, 30]. So obtaining more candidates still is important strategy for RORgt-targeted drug discovery in current stage in pharmaceutical industry. In this study, a set of lead compounds with tetraazacyclic moiety were identified to inhibit RORγt transcription activity, Th17 cell differentiation, and IL-17A secretion.

RORγt is involved in many physiological processes and widely expressed in thymus, brain, liver, muscle, pancreas, and other organs [31–33]. In addition to its T-cell related functions, RORγt is also involved in the regulation of phase I and phase II enzyme expression in liver, lipid and glucose metabolism, regulation of clock and circadian rhythm, and lymph node development [13]. Multiple functions of RORγt made it challenging to target its activity for therapy, as exhibited in the use of digoxin, which presents many side effects because of the multi-functionality of this pathway. Designing a T-cell specific drug will be a solution to this problem. In this study, we found that compounds 11 and 14 exhibited suppression of RORγt activity in T-cell derived cell-Jurkats and not in 293T cells, exhibiting potency that is cell type-specific. This variation in activity between two cell lines will provide the possibility to develop T cell specific drugs from these candidate compounds.

Recent studies have showed that the regulation of IL-17A and IL-17F expression by RORγt involves the CNS2 regulatory region at the il17-il17f locus [25, 34]. This indicates that IL-17A and IL-17F are direct targets of RORγt transcriptional regulation by binding to CNS2 region [34]. Our study also found that these screened candidates all exhibited significant inhibition on IL-17 expression at 5μM in the mice upon Th17 cell differentiation. Although compound 7, like SR1001, showed a relatively weak inhibition with ~50% suppression activity on IL-17A production, compounds 11 and compound 14 exhibited more potent inhibitory effect, with the reduction in IL-17A production close to 80% both in mRNA expression and protein secretion. However, compound 14 displayed no significant inhibition on IL-17F expression. The results suggested that these compounds have different inhibitory activities against IL-17A and IL-17F expression. Also we found that several compounds which inhibited only RORγt expression, rather than IL-17A and/or IL-17F expression (Fig 5). We proposal there are different transcript factors interacted with RORγt during regulation IL-17A and IL-17F transcription, and different compounds may take different effects on RORγt interacting with those transcript factors. As a result, they displayed different performs on RORγt expression and IL-17A expression, as well as IL-17F expression.

In addition, the three candidates, especially compound 11, displayed a high therapeutic efficacy with EC_50_ values at very low concentrations (nanomolar) in our Gal4-reporter system. Compounds 11 and 14 also displayed limited cytotoxicities in wild-type Jurkat cells, with CC_50_ values significantly greater than 5μM as determined by MTT assays. These results indicate that compounds 11 and 14 could be potent candidates with high T-cell specificity and limited cytotoxicity in Th17-mediated autoimmune diseases.

Structural analysis demonstrated that compounds 7, 11, and 14 share a highly similar scaffold. The three candidates are tetraazacyclic compounds, characterized by a tetrazolium benzene group and an aromatic heterocyclic group connected by a single sulfur bridge. However, these compounds demonstrated different effects on Th17 cell differentiation and RORγt activity. Our data showed that compound 7 could inhibit RORγt activity in 293T cells but the other two compounds cannot, compounds 7 and 11 dramatically inhibited IL-17F mRNA expression, but compound 14 had less effect on IL-17F expression in mouse T cells, although all compounds can inhibit IL-17A and RORγt expression well. These differences amongst three compounds indicate some structure-based effects which can inform future drug development. An analysis of SAR (structure-activity relationships) among these compounds is needed for optimization of these compounds for clinical use.

## Conclusion

In summary, we identified three tetraazacyclic compounds to be potent inhibitors of RORγt activity and IL-17A and IL-17F expression. These compounds also displayed limited cytotoxicity in cell-based assays. Moreover, two compounds exhibited T-cell specificity when compared with 293T cells. Furthermore, the tetraazacyclic scaffold, common among these compounds, can serve as a lead structure for future discovery of drug candidates in treating autoimmune diseases.

## Supporting Information

S1 File ARRIVE checklist form. (PDF)
